# Extracorporeal membrane oxygenation and acute kidney injury: a single-center retrospective cohort

**DOI:** 10.1038/s41598-023-42325-5

**Published:** 2023-09-13

**Authors:** Xiaolan Gao, Jacob Ninan, John K. Bohman, Jason K. Viehman, Chang Liu, Danette Bruns, Xuan Song, Xinyan Liu, Suraj M. Yalamuri, Kianoush B. Kashani

**Affiliations:** 1 Division of Nephrology and Hypertension, Department of Medicine, Mayo Clinic, 200 First Street SW, Rochester, MN 55905 USA; 2 Division of Life Sciences and Medicine, Department of Critical Care Medicine, The First Affiliated Hospital of USTC, University of Science and Technology of China, Hefei, 230001 Anhui China; 3 Division of Pulmonary and Critical Care Medicine, Department of Medicine, Mayo Clinic, Rochester, MN USA; 4 Division of Critical Care, Department of Anesthesiology, Mayo Clinic, Rochester, MN USA; 5 Division of Clinical Trials and Biostatistics, Mayo Clinic, Rochester, MN USA; 6 Department of Critical Care Medicine, Zhongnan Hospital of Wuhan University, Wuhan, 430071 Hubei China; 7 Anesthesiology Clinical Research Unit, Department of Anesthesiology, Mayo Clinic, Rochester, MN USA; 8 ICU, Shandong Provincial Hospital Affiliated to Shandong First Medical University, Shandong, China; 9 ICU, DongE Hospital Affiliated to Shandong First Medical University, Shandong, China

**Keywords:** Medical research, Nephrology

## Abstract

To assess the relationship between acute kidney injury (AKI) with outcomes among patients requiring extracorporeal membrane oxygenation (ECMO). This is a single-center, retrospective cohort study of adult patients admitted to intensive care units (ICU) at a tertiary referral hospital requiring ECMO from July 1, 2015, to August 30, 2019. We assessed the temporal relationship of AKI and renal replacement therapy with ECMO type (VV vs. VA). The primary outcome was in-hospital mortality rates. We used Kruskal–Wallis or chi-square tests for pairwise comparisons, cause-specific Cox proportional hazards models were utilized for the association between AKI prevalence and in-hospital mortality, and a time-dependent Cox model was used to describe the association between AKI incidence and mortality. After the screening, 190 patients met eligibility criteria [133 (70%) AKI, 81 (43%) required RRT]. The median age was 61 years, and 61% were males. Among AKI patients, 48 (36%) and 85 (64%) patients developed AKI before and after ECMO, respectively. The SOFA Day 1, baseline creatinine, respiratory rate (RR), use of vasopressin, vancomycin, proton pump inhibitor, antibiotics, duration of mechanical ventilation and ECMO, and ICU length of stay were higher in AKI patients compared with those without AKI (*P* < 0.01). While ICU and in-hospital mortality rates were 46% and 50%, respectively, there were no differences based on the AKI status. The type and characteristics of ECMO support were not associated with AKI risk. Among AKI patients, 77 (58%) were oliguric, and 46 (60%) of them received diuretics. Urine output in the diuretic group was only higher on the first day than in those who did not receive diuretics (*P* = 0.03). Among ECMO patients, AKI was not associated with increased mortality but was associated with prolonged duration of mechanical ventilation and ICU length of stay.

## Introduction

Extracorporeal membrane oxygenation (ECMO) is a cardiopulmonary support technology derived from intraoperative cardiopulmonary bypass machines. ECMO utilization rate continues to rise among patients with cardiovascular and/or respiratory failure^1–3^. Although ECMO is a life-saving therapy, it is associated with many complications, including acute kidney injury (AKI). The incidence of AKI among patients who require ECMO is reported to be about 50%^4^.

It has been reported that ECMO patients who develop AKI have unfavorable outcomes, particularly among more severe AKI requiring kidney replacement therapy (AKI-D). The hospital mortality rate in ECMO patients with AKI-D is 3.7 folds higher than those without AKI-D^5^. AKI is associated with a higher risk of mortality in ECMO patients, leading to higher morbidity and increased hospital costs^4,6^.

The association between AKI and ECMO outcomes has been vigorously investigated in recent years. The potential risk factors are the use of nephrotoxic drugs, hypoperfusion, bleeding, plasma-free hemoglobin levels, and duration of ECMO support^4,7,8^. However, the differences in patient outcomes based on the temporal relationship between AKI diagnosis and ECMO is not explored. In addition, there is a paucity of data related to the long-term outcomes of AKI among ECMO patients, chances of liberation from continuous renal replacement therapy (CRRT), and the impact of the type of ECMO support. Therefore, we aimed to explore these characteristics in this historical cohort of ECMO patients.

## Methods

### Subjects

All adult patients ≥ 18 years old who received ECMO support [venovenous (VV) or venoarterial (VA)] at Mayo Clinic, Rochester, Minnesota, from July 1, 2015, to August 30, 2019, were included. We excluded patients who did not provide Minnesota research authorization or were admitted to ICU while on ECMO from outside institutions. As our goal was to assess the relationship between AKI and ECMO, those with chronic, end-stage or acute kidney disease were excluded, including individuals who had end-stage kidney disease and received any dialysis modality within 14 days of admission. Only the first cannulation was considered for the type of support in patients with multiple cannulations. Patient data were abstracted from the electronic health records and the Mayo Clinic ICU database^9^. The Institutional Review Board (IRB) at Mayo Clinic, Rochester, Minnesota, has reviewed and approved this single-center retrospective cohort study. Obtaining informed consent was waived by Institutional Review Board (IRB) at Mayo Clinic, Rochester, Minnesota, due to retrospective nature of the study. All experiments were performed following relevant guidelines and regulations indicated by IRB.

We divided the cohort into three groups, (1) individuals with AKI before ECMO initiation, (2) patients who developed AKI after ECMO initiation, and (3) ECMO patients without AKI. Clinical outcomes were assessed, including mortality, chronic kidney disease, need for long-term dialysis, and kidney recovery. Patients without AKI were divided into two groups according to the type of ECMO support (VA vs. VV) to evaluate for additional mechanistic explanations. Those with AKI who required CRRT while on ECMO were divided into two groups: (1) CRRT machine connected to the ECMO circuit; (2) CRRT done conventionally with a separate dialysis catheter.

### Measurement

#### Definitions

AKI and its stages were adjudicated according to the KDIGO guidelines^10,11^. Patients with an increase in SCr by ≥ 0.3 mg/dl (≥ 26.5 µmol/l) within 48 h or an increase in SCr to ≥ 1.5 times baseline within seven days or urine output < 0.5 ml/kg/h for 6 h were included in the AKI cohort. Following AKI detection, we staged AKI using serum creatinine and urine output criteria from the KDIGO guidelines^10,11^. Supplemental Table 1 shows the AKI staging criteria. The baseline creatinine was calculated as the median of all the creatinine values within 180 days before the index ICU admission. In the absence of baseline serum creatinine levels, the back-calculation of serum creatinine using Modification of Diet in Renal Disease formula for GFR of 75 ml/min/1.73 m^2^^12^.

Time zero was the ECMO initiation time. Patients were followed until death or hospital discharge.

#### ECMO management

Mayo Clinic ECMO center follows the Extracorporeal Life Support Organization (ELSO; https://www.elso.org/Resources/Guidelines.aspx) guidelines for ECMO indications, equipment setup, and ECMO management parameters. Mayo Clinic primarily uses Cardiohelp ECMO consoles (Getinge AB) with HLS Set Advanced 7.0 disposable oxygenators. Alternatively, a CentriMag (Abbott) pump paired with a Quadrox-i oxygenator (Getinge AB) was also used. Our ECMO team closely monitors the ECMO circuit for evidence of thrombosis (visual inspection, post-oxygenator blood gas analysis, and to detect thrombolysis) and hemolysis (daily plasma free-hemoglobin levels and daily thromboelastogram complete blood counts).

#### Variables and outcomes

Patient demographics, clinical characteristics, hemodynamics, and laboratory data were abstracted from the electronic medical records. In addition, the type and parameters of ECMO support, mechanical ventilation, RRT, vasoactive drugs, and transfusion therapy were also collected. All enrolled patients were followed until discharge from the hospital, and the hospital survivors were followed for one year after discharge from the hospital.

The primary outcome was in-hospital mortality. Secondary outcomes included the safety and effect of diuretic use among those with AKI.

### Statistical analysis

Continuous data are presented as median (IQR), and categorical data are presented as frequency (percent). Pairwise comparisons were made for all variables to examine their association with AKI using Kruskal–Wallis or chi-square tests. The association between AKI prevalence (at ECMO initiation) and in-hospital mortality was assessed using cause-specific Cox proportional hazards models representing hazard ratios with 95% confidence intervals and p-values. The ICU mortality rates were presented using cumulative incidence curves with discharge as a competing risk. A time-dependent Cox model was used to describe the association between AKI incidence and mortality among those without prevalent AKI after adjusting for CCI, the severity of illness scores (APACHE and SOFA on day one of ECMO), lactate, baseline serum creatinine level, age, sex, and type of ECMO support (VV vs. VA). In the time-to-event analysis for AKI development after ECMO, time zero was defined as the time of ECMO initiation, with death as an event and hospital discharge as a censoring factor. Results were reported as hazard ratios with 95% confidence intervals and p-values.

Additionally, the cohort was divided into (1) AKI before ECMO initiation, (2) AKI developed during ECMO, and (3) without AKI. We conducted a cause-specific hazard model among patients in groups 2 and 3 by censoring patients at the ECMO completion or in-hospital death with AKI development as a dependent variable. Hazard ratios, 95% confidence interval, p-value, and Kaplan Meier curves were generated to assess the impact of the type of ECMO support (i.e., VV vs. VA) on the development of AKI. ECMO support type was defined as the initial setting, and crossover between VV and VA was not considered. Additionally, we evaluated the associations between the initial ECMO flow rate and pump speed on AKI development.

Among those who did not have chronic kidney disease before admission and were discharged alive, the frequency of de Novo CKD at 90 days and 1 year and the need for long-term dialysis were assessed. Among those with AKI requiring CRRT, the frequency of the CRRT liberation rate was calculated. In addition, the death rate after ECMO completion was described. Finally, we conducted a cause-specific hazards model among AKI patients who required CRRT to assess the death rate. CRRT initiation time was time zero for this analysis, and patients were censored at the time of ECMO liberation. A Kruskal–Wallis test was used for the patients in group 1 to compare urine output based on the diuretic challenge on days 1, 2, and 3.

### Ethical standard

Institutional Review Board (IRB) at Mayo Clinic, Rochester, Minnesota, has reviewed and approved this single-center retrospective cohort study. Due to the retrospective nature of the study, obtaining informed consent was waived by Institutional Review Board (IRB) at Mayo Clinic, Rochester, Minnesota. All experiments were performed following the 1964 Helsinki declaration and its later amendments for comparable ethical standards along with relevant national guidelines and regulations indicated by IRB.

## Results

**Figure 1:**
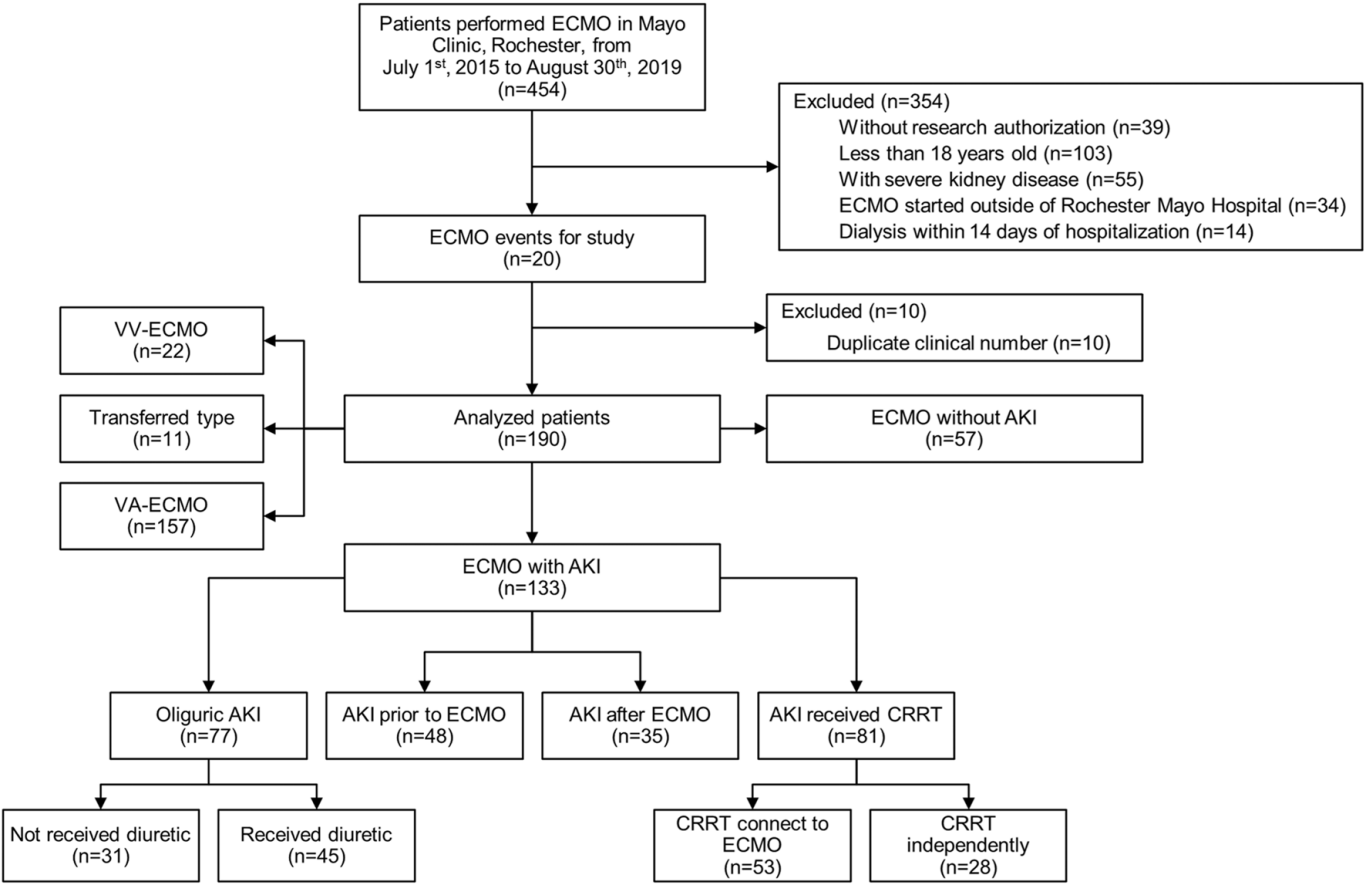
Patient flowchart.

Four hundred fifty-four patients who received ECMO were screened during the 4-year study period. The final analyses included one hundred ninety adult patients who met the eligibility criteria (Fig. 1). In this cohort, the median (IQR) age was 61 (47, 71), and 61% were male. The average estimated GFR (± SD) was 99.7 (± 13.8) ml/min/1.73 m^2^, with the lowest eGFR of 69.2 ml/min/1.73 m^2^. Of those, 133 (70%) had AKI, 157 (83%) received VA-ECMO, 22 (12%) were on VV-ECMO, and 11 (5%) crossed over from VV to VA during the study period. Among ECMO patients with AKI, 48 (36%) patients had AKI before ECMO initiation (group 1), 85 (64%) patients developed AKI after ECMO initiation (group 2), 77 (58%) had oliguric AKI, 46 (60%, n = 77) received the diuretic challenge, and 81 (61%) patients required RRT. Following excluding those who developed AKI before ECMO, the incidence of AKI was 45% (N = 91 out of 163). In a competing risk analysis with ICU death as a competing risk, the AKI incidence did not significantly differ (40%, Supplemental Fig. 1). Among those who received RRT, 53 (65%) used the ECMO circuit for RRT, and 28 (35%) had separate RRT central access (Fig. 1). The median (IQR) time on the need for the mechanical ventilator from the day ECMO was initiated was 0 (−1 to 0) days.

Table 1 shows the baseline demographic, clinical and laboratory data, and severity of illness before ECMO initiation. Among the included patients, 48 (25%) were in Group 1, 85 (45%) were in Group 2, and 57 (30%) had no AKI (group 3). The median age was 61 years, and 61% were males. The median respiratory rate (RR) was 17 (14, 22) breaths per minute among all patients, but it was significantly higher among those with AKI than those without AKI (20 (16, 22) bpm for prevalent AKI; 18 (14, 22) bpm for incident AKI;16 (12, 18) for no AKI; *p* < 0.01). Conversely, the temperature, heart rate, mean arterial pressure, and oxygen saturation were similar in all groups.

**Table 1:** Baseline characteristics and outcomes based on the timing of AKI.

Variables	Total	Prevalent AKI (Group 1)	Incident AKI (Group 2)		No AKI (Group 3)		*P*-value			
		n = 48	n = 85		n = 57		1 vs. 2	2 vs. 3	1 vs. 3	1 + 2 vs 3
Age, years	61 (47, 71)	56 (46, 69)	60 (44, 70)		64 (51, 71)		0.69	0.21	0.12	0.12
Male sex	115 (60.8)	35 (72.9)	56 (66.7)		24 (42.1)		0.46	< 0.01	< 0.01	< 0.01
CCI	4 (2, 6)	3.5 (2, 5)	4 (2, 5)		5 (2, 6)		0.69	0.1	0.052	0.05
APACHE3, 24 h	106 (76, 130)	118 (83, 138)	100.5 (82.5, 122)		84 (68, 128)		0.07	0.12	0.01	0.02
SOFA day 1	12 (9, 14)	13 (11, 14)	12 (10, 14)		10 (7, 14)		0.11	0.04	0.01	< 0.01
Baseline Cr, mg/dL	0.9 (0.7, 1.0)	0.8 (0.7, 0.9)	0.8 (0.7, 1.0)		1.0 (0.9, 1.1)		0.18	< 0.01	< 0.01	< 0.01
Vitals before ECMO										
Temperature, °C	36.5 (35.9, 37.0)	36.7 (36.3, 37.0)	36.6 (36.0, 37.0)		36.4 (35.6, 36.9)		0.67	0.28	0.15	0.43
HR, bpm	90 (77, 102)	90.5 (73, 108.5)	90 (79, 105)		88 (74.5, 99.5)		0.7	0.11	0.36	0.82
RR, bpm	17 (14, 22)	20 (16, 22)	18 (14, 22)		16 (12, 18)		0.19	< 0.01	< 0.01	< 0.01
MAP, mmHg	70.5 (53, 78)	69 (55.5, 74.5)	68 (51, 80)		73 (59, 82)		0.74	0.46	0.38	0.33
SPO_2_, %	96 (87, 100)	96 (87, 100)	96 (87, 100)		97 (84.5, 100)		0.83	0.98	0.77	0.77
Treatment during ECMO, N (%)										
Transfusion	180 (94.7)	45 (93.8)	81 (95.3)		54 (94.7)		0.7	0.88	0.83	0.05
Hypothermia	9 (4.7)	2 (4.2)	5 (5.9)		2 (3.5)		0.67	0.52	0.86	0.60
Dopamine	15 (7.9)	4 (8.3)	9 (10.6)		2 (3.5)		0.67	0.12	0.29	0.14
Norepinephrine	157 (82.6)	38 (79.2)	77 (90.6)		42 (73.7)		0.06	0.01	0.51	0.03
Epinephrine	160 (84.2)	42 (87.5)	74 (87.1)		44 (77.2)		0.94	0.12	0.17	0.08
Dobutamine	5 (2.6)	2 (4.2)	3 (3.5)		0		0.85	0.15	0.12	0.14
Vasopressin	150 (78.9)	41 (85.4)	74 (87.1)		35 (61.4)		0.79	< 0.01	0.01	< 0.01
Glycopeptides (vancomycin)	150 (78.9)	38 (79.2)	78 (91.8)		34 (59.6)		0.04	< 0.01	0.03	< 0.01
Aminoglycosides	9 (4.7)	2 (4.2)	6 (7.1)		1 (1.8)		0.5	0.15	0.46	0.20
Sulfonamides	27 (14.2)	10 (20.8)	13 (15.3)		4 (7.0)		0.42	0.14	0.04	0.06
Antivirals	15 (7.9)	7 (14.6)	6 (7.1)		2 (3.5)		0.16	0.37	0.04	0.26
Antifungals	6 (3.2)	2 (4.2)	4 (4.7)		0		0.89	0.10	0.12	0.10
NSAID	111 (58.4)	29 (60.4)	46 (54.1)		36 (63.2)		0.48	0.29	0.77	0.50
PPI	167 (87.9)	43 (89.6)	81 (95.3)		43 (75.4)		0.21	< 0.01	0.06	< 0.01
Radiocontrast	65 (34.2)	22 (45.8)	30 (35.3)		13 (22.8)		0.23	0.11	0.01	0.03
Phenylephrine	75 (39.5)	26 (54.2)	33 (38.8)		16 (28.1)		0.09	0.19	0.01	0.03
Mannitol	30 (15.8)	6 (12.5)	15 (17.6)		9 (15.8)		0.43	0.77	0.63	1
Allopurinol	7 (3.7)	1 (2.1)	3 (3.5)		3 (5.3)		0.64	0.61	0.42	0.45
Complications, N (%)										
DIC	23 (12.1)	8 (16.7)	10 (11.8)		5 (8.8)		0.43	0.57	0.22	0.36
Hemolysis	25 (13.2)	11 (22.9)	9 (10.6)		5 (8.8)		0.06	0.72	0.04	0.24
Thrombus	73 (38.4)	19 (39.6)	34 (40)		20 (35.1)		0.96	0.55	0.63	0.54
Bleeding	100 (52.6)	26 (54.2)	45 (52.9)		29 (50.9)		0.89	0.81	0.74	0.75
Microembolisms or atheroembolic disease	3 (1.6)	0	2 (2.4)		1 (1.8)		0.28	0.81	0.36	0.89
Neurological events	39 (20.5)	9 (18.8)	19 (22.4)		11 (19.3)		0.62	0.66	0.94	0.78
Infection	35 (18.4)	14 (29.2)	16 (18.8)		5 (8.8)		0.17	0.1	0.01	0.03
Coagulopathy	128 (67.4)	31 (64.6)	58 (68.2)		39 (68.4)		0.67	0.98	0.68	0.84
Duration, days										
ECMO duration	3.8 (1.9, 8.3)	6.6 (2.3, 13.0)	4.6 (2.5, 8.2)		2.5 (0.9, 4.3)		0.22	< 0.01	< 0.01	< 0.01
MV duration	8.8 (3.2, 20.3)	10.8 (3.9, 27.2)	13.1 (5.2, 24.2)		3.9 (2.1, 7.5)		0.67	< 0.01	< 0.01	< 0.01
Length of ICU stay	9.2 (4.7, 18.8)	15.0 (7.3, 23.6)	10.9 (6.1, 20.1)		5.2 (2.6, 10.3)		0.27	< 0.01	< 0.01	< 0.01
Length of hospital stay	19.3 (8.9, 37.7)	23.3 (9.6, 41.5)	21.1 (13.2, 46.9)		15.1 (5.9, 24.3)		0.94	< 0.01	0.01	< 0.01

*CCI* Charlson Comorbidity Index, *APACHE* Acute Physiology, and Chronic Health Evaluation, *SOFA* Sequential Organ Failure Assessment, *ECMO* extracorporeal membrane oxygenation, *HR* heart rate, *RR* respiratory rate, *MAP* mean arterial pressure, *SPO*_*2*_ oxygen saturation by pulse oximetry, *IVIG* intravenous immunoglobulin, *NSAIDs* non-steroidal anti-inflammatory drugs, *PPI* proton pump inhibitor, *DIC* diffuse intravascular coagulation, *ICU* intensive care unit, *MV* mechanical ventilation.

In the whole cohort, 180 (95%) received blood products, 9 (5%) received targeted hypothermia, and 5 (3%) ambulated during ECMO. The use of epinephrine (160, 84%), norepinephrine (157, 83%), vasopressin (150, 79%), vancomycin (150, 79%), proton-pump inhibitors (167, 88%), and nonsteroidal anti-inflammatory drugs (111, 58%) was common. Groups 1 and 2 (85% and 87%, respectively) received more vasopressin than group 3 (61%) (*p* < 0.05). The usage of quinolones (23, 12%), radiocontrast (52, 27%), and phenylephrine (59, 31%) amongst patients on ECMO with AKI was higher than those on ECMO without AKI (*p* < 0.05).

The in-hospital mortality rate among ECMO patients was 50%, with most deaths occurring in the ICU (46%) (Table 1). The incidence of Disseminated intravascular coagulation (DIC), hemolysis (defined as progressive anemia with laboratory evidence of anemia), thrombotic events, bleeding, microembolisms, neurological accidents (i.e., ischemic or hemorrhagic stroke), infection, and coagulopathy were 12%, 13%, 38%, 53%, 2%, 21%, 18, and 67%, respectively. There were no statistical differences amongst the three groups for the above complications, but ECMO with AKI group had a higher risk of infection than ECMO without AKI (*P* < 0.05).

**Figure 2:**
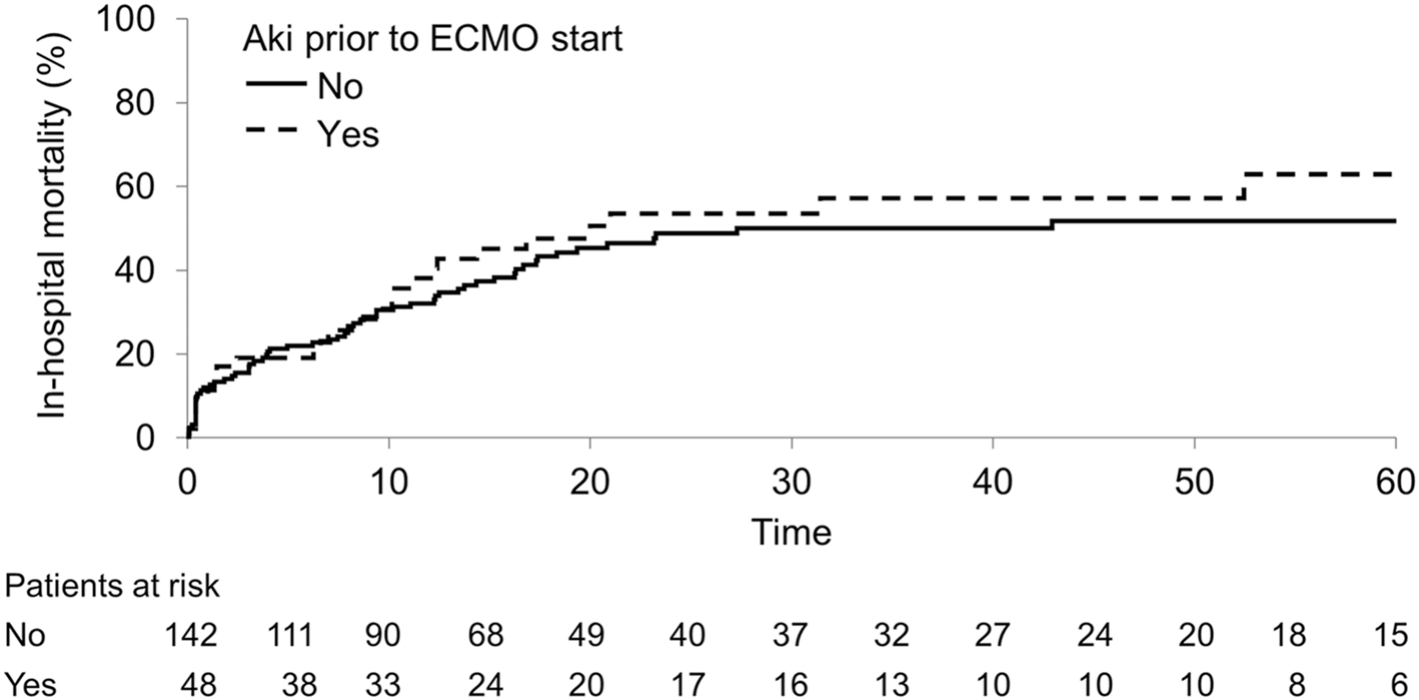
In-hospital mortality based on groups.

The duration of mechanical ventilation was longer in groups 1 and 2 compared with group 3 (11 (4, 27) and 13 (5, 24) vs.4 (2,8), *P* < 0.01). The ICU length of stay was longer in groups 1 and 2 than group 3 (15 (7, 24) and 11 (6, 20) vs. 5 (3, 10), respectively, *P* < 0.01). We found no differences in mortality between groups in any analyses done (Fig. 2 and Table 2).

**Table 2:** The association between groups and in-hospital mortality.

Model	HR (95% CI)	*P*-value
AKI before ECMO (Group 1) (compared to groups 2 and 3)	1.30 (0.81, 2.09)	0.278
Time-dependent AKI (acquired during ECMO)	1.13 (0.51, 2.52)	0.756

This table includes two separate models. The first model compares in-hospital mortality among patients with AKI before ECMO (group 1) to the other groups. The second shows the risk of in-hospital mortality after developing AKI in the subset of patients that did not have AKI before ECMO.

The incidence of de Novo CKD among those survivors and ECMO-liberated patients was relatively modest (~ 10% at 90 days and 1-year follow-up). Among those who developed AKI requiring CRRT, 25 (64%) were liberated from CRRT due to improvement in kidney function, 12 (31%) withdrew CRRT due to a change in goals of care, and 2 (5%) stopped CRRT for other reasons (Table 3). The association with in-hospital mortality with groups was not significant (prevalent AKI as the reference; incident AKI HR 1.05 [95%CI 0.34–3.20] and no AKI HR 0.65 [95%CI 0.20–2.15]; Supplemental Table 2). There were no statistical differences in clinical outcomes of those who developed AKI before or after ECMO initiation (Table 4).

**Table 3:** Frequency of de Novo CKD in survivors and ECMO-liberated.

Outcomes	N = 97	%
90 day CKD status, n (%)		
No	87	90
Yes	10	10
1 year CKD status, n (%)		
No	86	89
Yes	11	11
Long-term dialysis, n (%)		
No	92	95
Yes	5	5
AKI requiring CRRT	39	40
Reason for withholding CRRT, n (%)		
Improved	25	64
Withdrawal, death	12	31
Other	2	5

**Table 4:** Outcomes based on the temporal relationship between AKI and ECMO.

	AKI before ECMO initiation (n = 48)	AKI after ECMO initiation (n = 85)	P-value
Oliguric AKI, N (%)	25 (52)	50 (59)	0.5
Oliguric AKI received diuretics, N (%)	17 (35)	29 (64)	0.9
Need for CRRT, N (%)	30 (63)	51 (60)	0.8
CRRT duration, days	18 ± 36	25 ± 102	0.6
CRRT connected to ECMO, N (%)	17 (57)	36 (71)	0.4
Liberated from CRRT, N (%)	46 (96)	82 (97)	0.9
CKD prior to ECMO, N (%)	7 (15)	10 (12)	0.6
CKD 90, N (%)	10 (21)	10 (12)	0.2
CKD 1 year, N (%)	9 (19)	11 (13)	0.4
Long-term dialysis, N (%)	2 (4)	3 (4)	0.9

**Figure 3:**
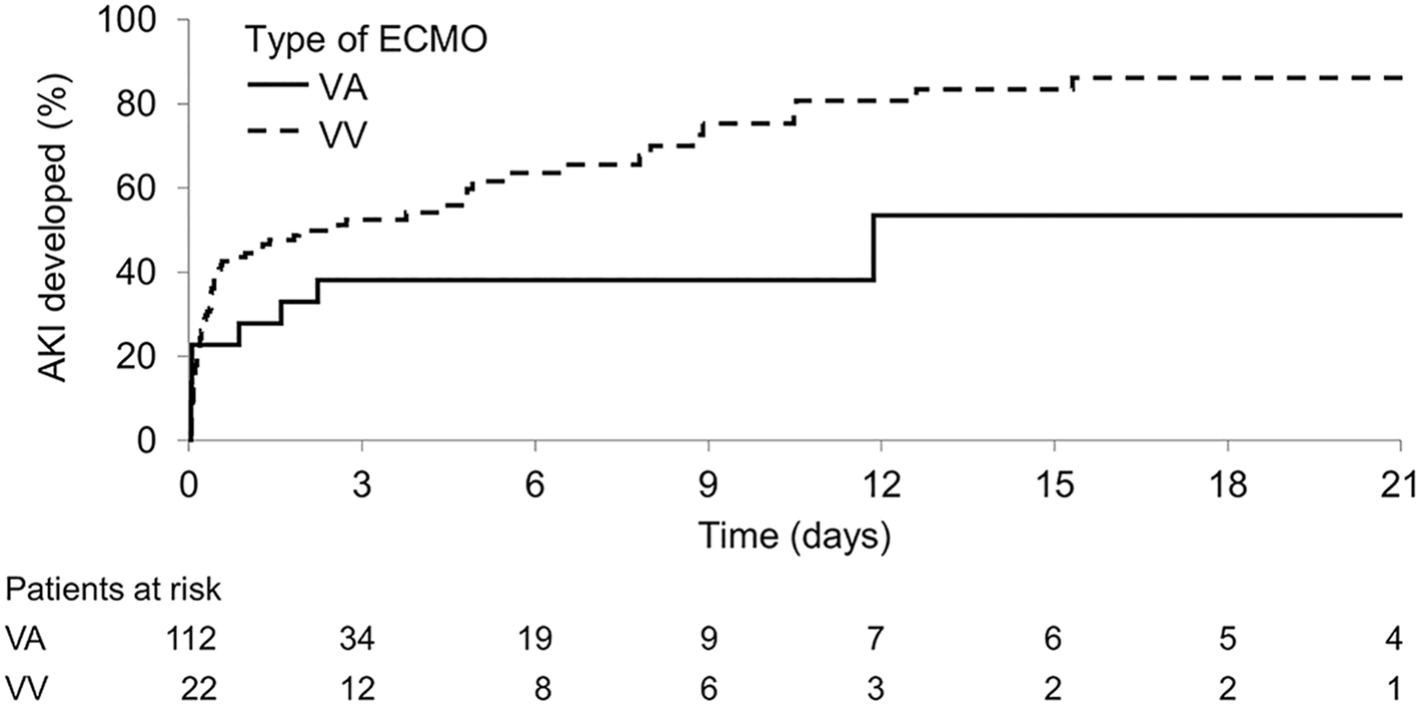
The association between AKI and ECMO based on the type of ECMO support (VV *vs.* VA).

ECMO support type, i.e., VV vs. VA, did not impact the development of AKI (HR 0.52, 95%CI 0.27 to 1.02, *P* = 0.06; Fig. 3 and Table 5). There were no associations between the mean ECMO blood flow rate and pump speed on day 1 with AKI (HR 1.10, 95%CI 0.84–1.45, *P* = 0.5, and HR per 100 RPM 1.13, 95%CI 0.51–2.52, *P* = 0.7, respectively; Supplemental Table 3). There were no statistically significant differences in the hospital mortality rate based on CRRT access (HR 1.77, 95%CI 0.72–4.35, *P* = 0.2; Supplemental Fig. 2 and Supplemental Table 4).

**Table 5:** The risk of AKI based on ECMO type.

Variable	HR (95% CI)	*P*-value
VV ECMO	0.52 (0.27, 1.02)	0.06
VA ECMO	Reference	

The urine output on day one was higher in patients who received diuretics than those who did not (*P* = 0.03; Table 6). However, urine output was not statistically different based on diuretic utilization after the first day of ECMO (*P* > 0.05). In addition, the mortality did not change based on diuretic administration (HR 0.90, 95%CI 0.42–1.92, *P* = 0.8).

**Table 6:** The association between initiation of diuretics and urine output in oliguric AKI patients.

	Diuretics			
	No (N = 22)	Yes (N = 37)	Total (N = 59)	*P*-value
Day 1 urine output, median (IQR)	833.0 (388.0, 2427.0)	1901.0 (1080.5, 2770.9)	1498.0 (786.0, 2709.0)	0.03
Day 2 urine output, median (IQR)	407.0 (39.0, 3147.0)	1406.0 (789.0, 2544.0)	1165.0 (133.0, 2923.0)	0.16
Day 3 urine output, median (IQR)	599.0 (49.0, 3262.0)	1618.0 (36.0, 2919.0)	1410.0 (36.0, 3090.5)	0.58
In-hospital death, hazard ratio (95% CI)	Reference	0.90 (0.42, 1.92)		0.78

## Discussion

This comprehensive assessment of a large cohort of ECMO patients and AKI in a tertiary hospital demonstrates a very high incidence of AKI (70%) and AKI requiring dialysis (43%) among ECMO patients. Moreover, we found no significant correlations between AKI and in-hospital mortality after adjustments for CCI, the severity of illness, lactate, baseline serum creatinine level, age, sex, and type of ECMO support. Of our secondary outcomes, the duration of mechanical ventilation and ICU length of stay were significantly higher among patients with AKI than those without AKI. We found a clinically relevant but statistically insignificant higher risk of AKI among those on VV-ECMO compared to VA-ECMO (HR 0.52, *P* = 0.06). The statistical insignificance could be due to type II error secondary to a smaller sample size. The trend mentioned above may further indicate the need to investigate this particular AKI risk factor. The ECMO settings, including flow rate and pump speed, were not associated with AKI risk. However, this analysis is limited because only the initial settings were considered. Diuretics were safe and were associated with improvement in urine output only on the first day. Higher RR, usage of vasopressin, vancomycin, PPI, quinolones, radiocontrast, and phenylephrine administration were more common among AKI patients. The temporal relationship between AKI and ECMO did not impact CRRT liberation rate, CKD at 90 days, and one year.

### Comparison with previous studies

With the progressive ECMO utilization in the past decade, the AKI incidence has been increasingly reported as 30–70%^5,13–17^. This is substantially higher than other etiologies of AKI in intensive care units^18–20^. The pathophysiology of AKI among ECMO patients is quite complex and mostly remains unclear. The potential AKI risk factors are low cardiac output syndrome, exposure to nephrotoxic agents (i.e., vasoactive drugs, antibiotics, radiocontrast), new-onset sepsis, fluid overload, ischemia–reperfusion injury, proinflammatory mediators release, oxidative stress, hemolysis, hemorrhage, and/or thrombosis^21–25^. A recent meta-analysis demonstrated that AKI incidence among VA-ECMO patients (60.8%) was higher than that of VV-ECMO (45.7%)^5^. Our study found that the risks were similar, but the small sample size of VV-ECMO patients could have influenced the result. One of the main differences between the two modalities is that VA-ECMO provides smaller pulse pressure while VV-ECMO maintains pulsatile cardiac output^26^. Pulsatile flow may protect microcirculation and kidney perfusion^27,28^.

Lee et al. observed a lower AKI rate with higher ECMO pump speed^29^. In contrast, increased ECMO flow rate and pump speed on day one were not associated with AKI risk. One limitation of our analysis is that only the settings on day one were considered. Therefore, prospective studies are required to more thoroughly assess the effects of ECMO pump speed on AKI risk in ECMO patients.

In-hospital mortality in AKI patients is nearly twice that of those without AKI^30^. In contrast, in our study, in-hospital mortality was similar amongst the groups. Furthermore, after adjustment for confounders, we found no differences in mortality among those with prevalent AKI, incidental AKI, and no AKI. Our findings differ from a recent study that showed AKI as an independent risk factor for mortality in patients requiring ECMO^31^. The reason for this difference could be variabilities in sample size. Thus, a more extensive multicenter study may be needed for further clarification.

An earlier study reported that diuretics in critically ill patients with acute kidney injury were associated with an increased risk of death. Another investigation focused on administering diuretics in oliguric AKI patients on ECMO^32^ indicated that more than half of ECMO patients with AKI were oliguric, and up to 60% received a diuretic challenge. We compared the urine output during the first 3 days between those with and without diuretic administration. We noted that urine output responded to diuretics only on day 1 and did not impact mortality rates. This demonstration may indicate that diuretic administration is likely safe and effective if used earlier during ECMO support.

RRT requirement is common among ECMO patients with severe AKI^33–35^. Recent studies compare the advantages and disadvantages of RRT initiation and the direct connection of RRT to a central venous catheter vs. connection to the ECMO circuit^16,36–41^. In a recent systematic review, Ostermann et al.^42^ concluded that the mortality risk is not dependent on the methods of combining ECMO and RRT. In our study, 35% of RRTs were done via a central venous catheter and 65% via the ECMO circuit. The type of RRT connection was not associated with mortality. While connecting CRRT to the ECMO circuit can avoid additional catheter-associated complications, it could be associated with adverse events, such as ECMO circuit clotting, pressure alarms, and the inability to control the net ultrafiltration rates^43,44^.

Recently, a large-scale survey was conducted in China about drug-induced hospital-acquired AKI. Anti-infectives, diuretics, and proton pump inhibitors were the top 3 drug classes associated with AKI risk^45^. Despite the potential nephrotoxicity of vancomycin, given its efficacy against gram-positive cocci, especially MRSA, its use among ECMO patients may be inevitable. There are also investigations highlighting PPI usage was associated with adverse kidney outcomes and significantly increased the risks of AKI^46–49^. Our study indicated that incidental AKI was significantly more common when PPIs were administered (*P* < 0.01). Therefore, reducing PPI use or shortening its utilization could decrease AKI risk.

We found vasopressin utilization was more common among incidental AKI patients (*P* < 0.01), possibly due to the higher severity of illness, indicating a higher risk of AKI and more hemodynamic instability. In clinical trials^50–54^, vasopressin reduced AKI development and progression and lowered mortality in patients with septic shock or after major procedures. Vasopressin, vancomycin, and PPIs may be independent predictors of AKI among ECMO patients. Among drugs used in this cohort, NSAIDs were used among 58% of patients. Based on our protocols, while NSAIDs are recommended over opioids for low-risk patients, when AKI risk increases, they are discontinued. In our cohort, almost all NSAIDs used during the index admission were before the need for ECMO or increased AKI risk.

This study possesses several strengths. First, we comprehensively evaluated AKI risk factors and impacts among ECMO patients in a large cohort. Second, we compared prevalent and incidental AKI (AKI before and after ECMO initiation) with each other and with those without AKI. Furthermore, we assessed the association of ECMO strategies with AKI and clinical outcomes. Finally, we included 1-year follow-up to assess the long-term major outcomes of ECMO and AKI. However, our study possesses several limitations. First, this study was a single-center retrospective study subject to all associated biases common to historical cohort studies. Due to the study design, we cannot assert a causal relationship in our findings, and the results only indicate correlations for hypothesis generation. Second, all included patients were adults, and findings cannot be extended to the pediatric population. Third, the sample size, particularly VV-ECMO, was low. In addition, information regarding the ECMO indication was missing from our database. Finally, as we excluded patients with CKD, future studies may need to explore the role of chronic kidney disease in the relationship between ECMO and AKI.

## Conclusion

AKI among ECMO patients is very common. As ECMO patients' mortality is often related to cardiorespiratory failure or other ECMO-related complications, we did not find any association between AKI and mortality in this cohort. Hospital length of stay and duration of mechanical ventilation were longer in those with AKI than in those without AKI. All of these findings need further prospective investigations.

### Supplementary Information

Supplementary Information 1.

Supplementary Figure 1.

Supplementary Information 2.

## Data Availability

All data generated or analyzed during this study are included in this published article and its supplementary information files.
