# Supplementary figures and images for: Extracorporeal membrane oxygenation and acute kidney injury: a single-center retrospective cohort
Source: Sci Rep. 2023 Sep 13;13:15112. doi: 10.1038/s41598-023-42325-5 (PMC10499785; doi:10.1038/s41598-023-42325-5)

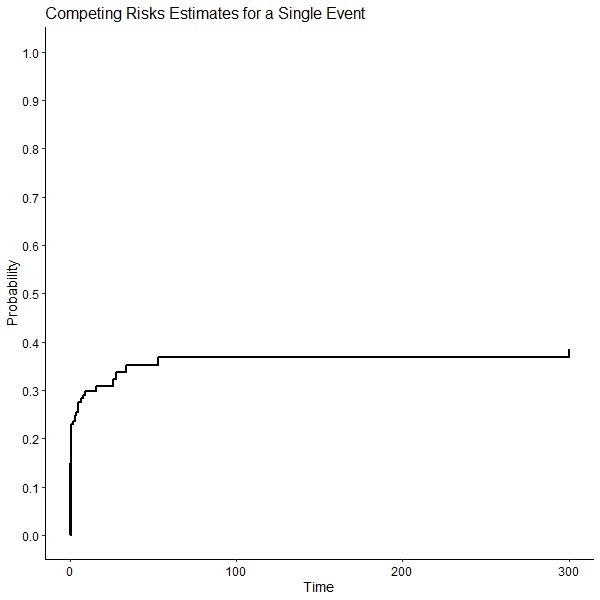

Supplement: Supplementary file 2 — Supplementary Figure 1. [file 41598_2023_42325_MOESM2_ESM.tif]
