# Supplementary material for: Extracorporeal membrane oxygenation and acute kidney injury: a single-center retrospective cohort
Source: Sci Rep. 2023 Sep 13;13:15112. doi: 10.1038/s41598-023-42325-5 (PMC10499785; doi:10.1038/s41598-023-42325-5)

| Supplemental Table 1 KDIGO staging criteria for AKI | | |
| --- | --- | --- |
| Stage | Serum creatinine | Urine output |
| 1 | 1.5–1.9 times baseline  OR  ≥0.3 mg/dl (≥26.5 µmol /l) increase | <0.5 ml/kg/h for 6–12 hours |
| 2 | 2.0–2.9 times baseline | <0.5 ml/kg/h for ≥12 hours |
| 3 | 3.0 times baseline  OR  Increase in serum creatinine to ≥4.0 mg/dl (≥353.6 µmol /l)  OR  Initiation of renal replacement therapy | <0.3 ml/kg/h for ≥24 hours  OR  Anuria for ≥12 hours |

| Supplemental Table 2: The association between in-hospital mortality with AKI prevalence or incidence | | |
| --- | --- | --- |
| Variable Level | HR (95% CI) | Overall p-value |
| AKI before ECMO | Reference | 0.7 |
| AKI after ECMO | 1.05 (0.34, 3.20) |  |
| No AKI | 0.65 (0.20, 2.15) |  |

| Supplemental Table 3: The association of ECMO Flow rate and pump speed with AKI development | | |
| --- | --- | --- |
| Variable | HR (95% CI) | p-value |
| Mean Day 1 Flow, per 1 | 1.10 (0.84, 1.45) | 0.5 |
| Mean Day 1 RPM, per 100 | 1.01 (0.98, 1.04) | 0.7 |

| Supplemental Table 4: Relationship between CRRT access and survival | | |
| --- | --- | --- |
| CRRT access | HR (95% CI) | p-value |
| ECMO circuit | 1.77 (0.72, 4.35) | 0.2 |
| Independent dialysis catheter | *Reference* |  |

**Supplemental Figure 1:** AKI incidence after accounting for ICU death as a competing risk


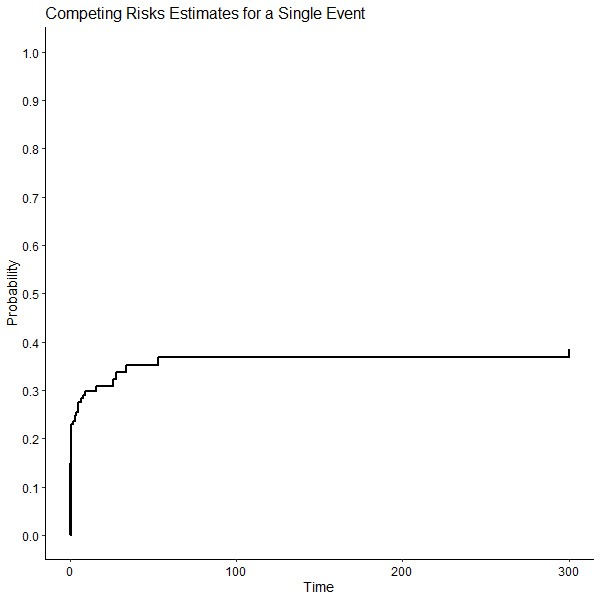


**Supplemental Figure 2:** Relationship between CRRT access and survival


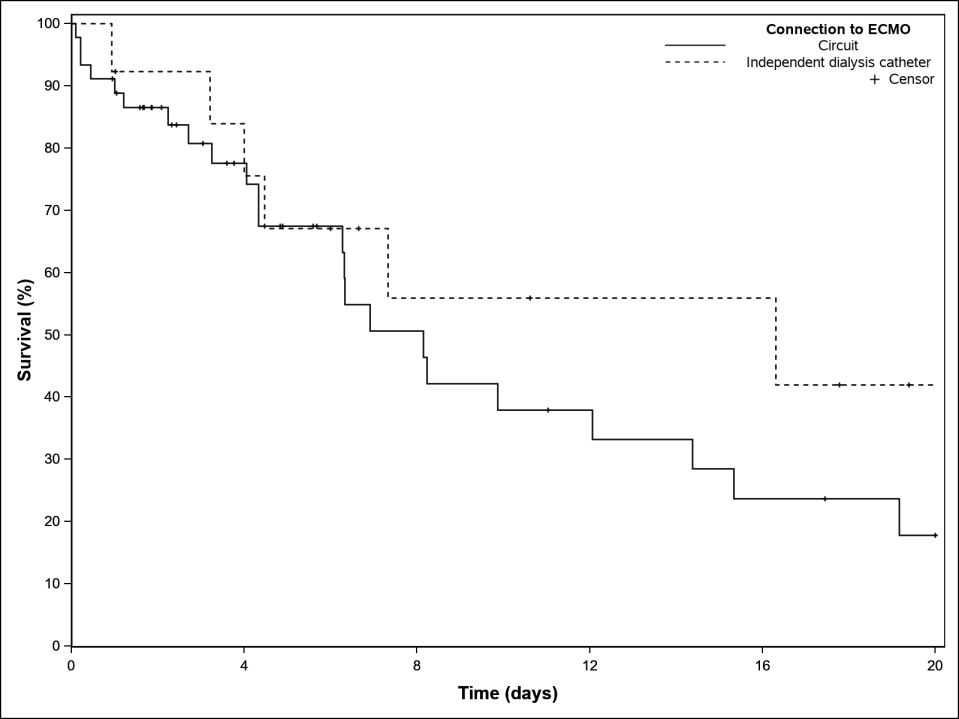

Supplement: Supplementary file 3 — Supplementary Information 2. [file 41598_2023_42325_MOESM3_ESM.docx]
